# Development of defibrillation simulator with LC-Tank type inductance coupling position measurement system

**DOI:** 10.1371/journal.pone.0214576

**Published:** 2019-03-29

**Authors:** Young Seok Lee, Su Hwan Oh, Yong Suk Lee, Ae-Young Her, In Bae Chang

**Affiliations:** 1 Department of Mechanical and Mechatronic Engineering, Kangwon National University, Gangwondaehak-gil, Chuncheon-si, Gangwon-do, South Korea; 2 School of Medicine, Kangwon National University, Gangwondaehak-gil, Chuncheon-si, Gangwon-do, South Korea; Texas A&M University College Station, UNITED STATES

## Abstract

In this study, we construct a paddle positioning system for defibrillation training simulators. The system consists of a chest manikin fabricated based on the standard somatotype of Koreans and simulator paddles similar to those of commercial defibrillators. Multiple coils are arranged orthogonally in both the chest manikin and paddles; their positioning is based on electromagnetic induction, such that the positions of the paddles can be successfully detected across the chest. The calculated and actual positions are compared using the measured signals. We find a mean error of 0.0891 mm for all positions, with a standard deviation of 0.6611 mm. In addition, we connect our paddle positioning system to a graphical user interface that enables visualization of the paddles’ current locations.

## Introduction

**Fig 1:**
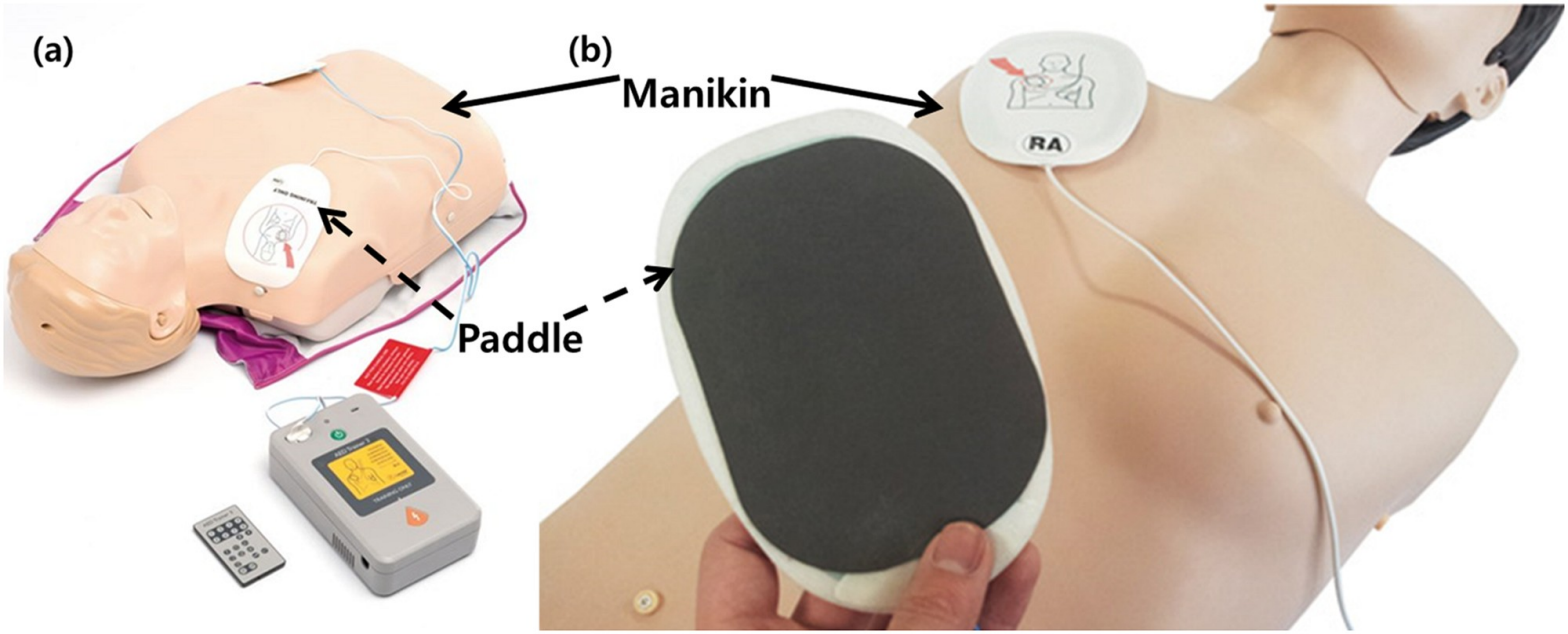
Example of CPR training simulators. (a) Laerdal–Anne; (b) BT–CPTA; Typical AED training devices are chest models and paddles as shown, which are mainly devices that include CPR and defibrillation related training functions.

The spread of western-style diet and lifestyle changes has resulted in a gradual increase in heart disease patients in Korea; in particular, the number of heart attack patients and deaths has consistently increased [1]. A heart attack is highly fatal when not medically treated, and although heart attack survival rates differ significantly between in-hospital cardiac arrest patients (IHCAs) and out-of-hospital cardiac arrest patients (OHCAs), appropriate first aid can save many more patients. The American Heart Association (AHA) provides guidelines for treating cardiac arrest patients [2]. If a person is under cardiac arrest outside hospital grounds, cardiopulmonary resuscitation (CPR) must be conducted along with an immediate call for rescue. Meanwhile, an automatic external defibrillator (AED) should be secured and used as soon as possible. If a cardiac arrest occurs in a hospital, a defibrillator must be prepared while CPR is carried out on the patient. Defibrillation is an essential treatment for cardiac arrest patients, and the AHA guidelines also include the methodology for increasing defibrillation’s success rate. For effective defibrillation, firstly, paddles should be placed in effective positions; secondly, adequate pressure should be applied to the paddles. These two tips are crucial to the effective delivery of electrical energy to a heart. However, recent research has revealed that a considerable percentage of medical personnel have faced difficulty in correctly placing defibrillator paddles [3,4]. Fortunately, numerous educational defibrillation techniques are commercially available; Fig 1 shows several commercial simulators for emergency rescue training that can reproduce diverse emergency situations [5,6]. CPR and defibrillation training functions include the simulation of cardiac arrest; however, most existing simulators that provide defibrillation training functions focus on analyzing heart rhythms to determine whether defibrillation should be conducted. These devices only check the attachment of electrode paddles or the polarity inversion, and signals are identified by attaching the paddles to electrode extrusions before carrying out defibrillation. Accordingly, defibrillation training is ineffective and training environments are unrealistic. This study establishes a system for training defibrillation techniques that provides a realistic environment and enables practice of correct paddle positioning.

## Materials and methods

To construct a defibrillation training system in which realistic paddle positioning can be practiced, the following requirements must be fulfilled:

1. Human chest manikins and defibrillator paddle simulators must be realistic;
2. A measurement system must identify the positions of paddles attached to the chest manikin;
3. The system must be compatible with commercial defibrillators.

However, the common defibrillator is not satisfied as the above conditions. Therefore, this paper deals with the development of a defibrillation simulator that satisfies the following conditions:

1. The chest manikin must cover the full defibrillation range of commercial defibrillator paddles;
2. There should be no electrodes or other devices on the surface of the chest manikin;
3. The paddles’ measurement coverage on the chest manikin surface must abide by the recommended paddle positions of the AHA’s defibrillation guidelines.

In addition to the above conditions and restraints, the development of this training tool also involved building a positioning system that shows the current locations of paddles on the chest manikin. This system must use a contactless positioning sensor to measure locations without attaching anything to the surface of the chest manikin. However, conventional position sensors, such as linear sensors and laser sensors, are not suitable for situations where measurement is carried out non-contact with the surface of the manikin forming an irregularly curved surface. Accordingly, a new type of positioning system needed to be devised. One alternative consisted of installing a Hall sensor inside the chest manikin. A Hall Effect sensor (such as US1881-latch type, US5881-Non latch type) can identify the presence of a magnetic field using a simple integrated circuit (IC). If a Hall sensor is placed below the skin of the chest manikin and magnets are attached to paddles, the positions of paddles can be measured. However, the number of necessary sensors may increase depending on the positioning area and resolution required, which could also increase the number of input/output (I/O) ports of a microcontroller unit (MCU). This study was suggested and develops a new method to overcome the above problem and accomplish the goals.

**Fig 2:**
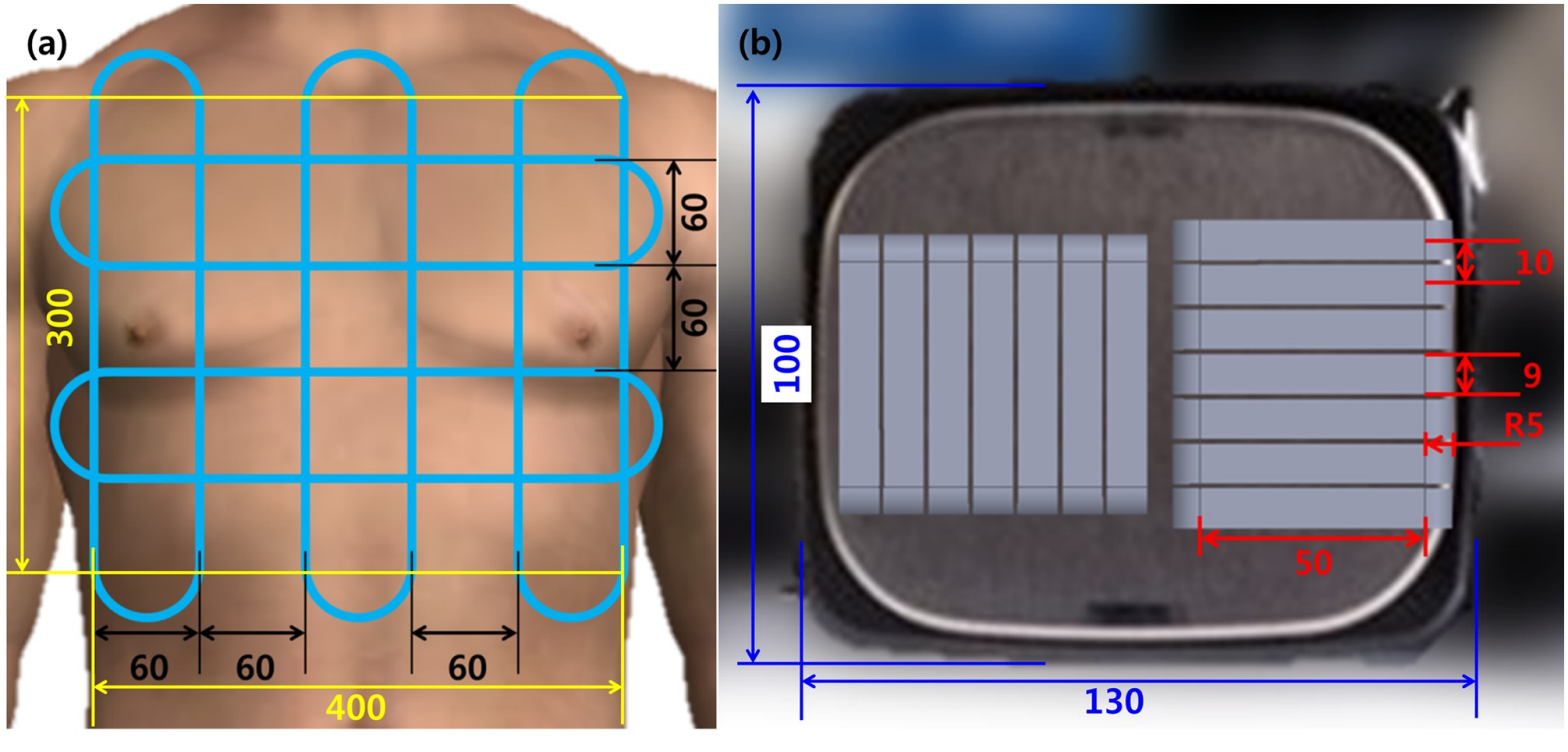
Size and coils placement of chest manikin and defibrillator positioning paddle. (a) chest manikin; (b) paddle.

First, the size of the chest manikin for the defibrillation training system needed to be determined based on the requirement that the left paddle is attached around the sternum and the right one at the apex of the heart. The average size of the Koreans in their 20s, which was provided by Size Korea, a Korean standard human size research institute, was considered; the model’s final size was determined as shown in Fig 2a [7,8]. The area of the electrode plates of commercial AED paddles was applied to our simulator; the actual measurements are illustrated in Fig 2b [9]. This study used electromagnetic induction to measure locations in a 400 mm × 300 mm area. Receiving coils were arranged in the chest manikin and transmitting coils were placed in the paddles. Voltages were induced to the receiving coils by driving the transmitting coils and the induced voltages were measured. An LC-tank resonance circuit (is configured merely with inductor and capacitor) was configured in the receiving coils to improve the selectivity of the transmittance frequency and compensate for the low coupling multiplier of an air-cored coil, thereby facilitating voltage measurement. If the system were configured by receiving and transmitting units, each consisting of a single coil, the required positioning measurement area could not be effectively covered. Therefore, in this study, we have organized the coil layout to achieve the goal. The receive coil was fabricated in the same shape as the solid blue line in Fig 2a and placed in the chest model. As shown in Fig 2b, the transmission coil is placed so that the center axis of the coil is parallel to the electrode surface of the paddle. The transmission coil is arranged in seven horizontal and vertical directions for each paddle. Below are the specifications and arrangement of the coils that make up the system.

Fig 2a shows the configuration of the receiving coils. To cover the whole chest area, three coils and two coils were arranged in the transverse and longitudinal directions, respectively. Coils had a central width of 60 mm and a winding width of 3 mm. Each coil had 200 windings of 0.3-mm enamel wire. The space between coils (the distance from the center of one coil to that of the next) was set to 120 mm. Based on these specifications, the receiving coils were arranged as shown in Fig 2a. Fig 2b shows the configuration of transmitting coils. Seven transmitting coils were arranged in both the transverse and longitudinal directions. Coils had a straight groove shape, with an inner diameter of 10 mm and a winding width of 9 mm. The space between coils was set to 1 mm; thus, the width space was planned to be 10 mm. Each coil had 50 windings with 0.3-mm enamel copper wire.

**Fig 3:**
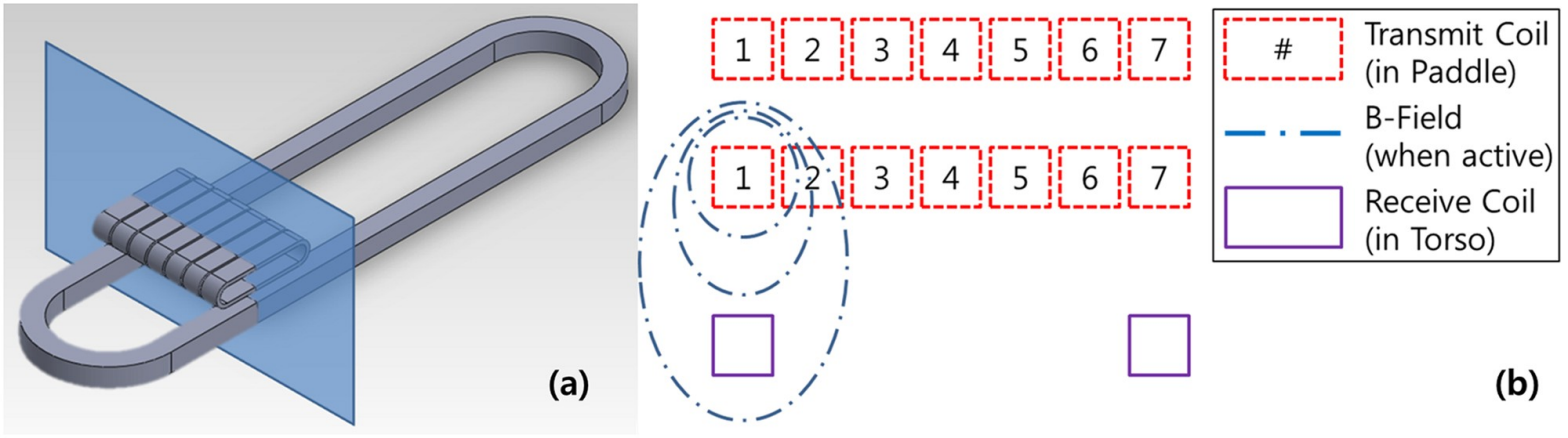
Concept of position measurement system. (a) arrangement of the receiving and transmitting coils; (b) concept of position measurement using inductance coupling.

Fig 3a illustrates the arrangement of the receiving and transmitting coils. The large loop at the bottom indicates coils arranged along the plane of the chest manikin and the multiple small loops in the upper part of the figure denote the transmitting coils installed in the paddles. Fig 3b is a cross-section cut in the direction of the blue plane of the coil, arranged as shown on the left. When transmitting coils are driven, a Magnetic Field was generated by current, which induced current on the receiving coils arranged below.

**Fig 4:**
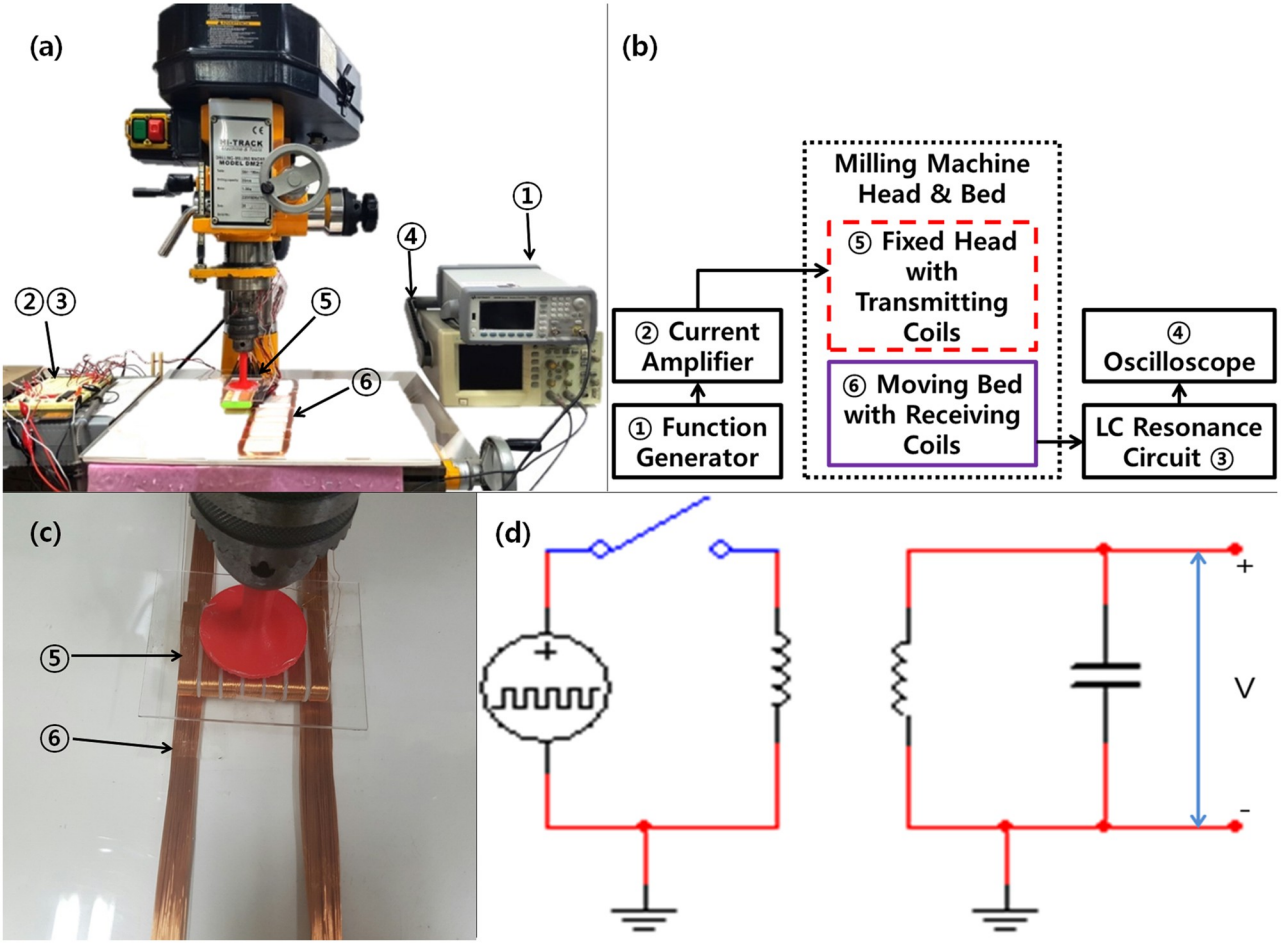
Developed experimental device that is measured position detection performance. (a) experimental device consist of manual milling machine, function generator, oscilloscope and auxiliary electrical circuitry (b) schematic diagram of system configuration (Fig 4a) (c) transmitting coil align with receiving coils (d) briefly drawn electrical circuit of experimental device.

The transmitting and receiving unit are connected by inductance coupling, as is common for capacitance sensors [10–12]. In the case of inductance coupling of a circular coil, the induced voltage value according to the distance between the transmitting coil and the receiving coil can be obtained through calculation. But in this study, coils are not a circle and not equal size. Therefore, it is difficult to derived inducing voltage by calculation. Thus, the value of the induced voltage can be obtained according to the distance between transmitting and receiving coils by calculating impedance. However, coils’ shape is not circular and transmitting and receiving coils have a different dimension. Accordingly, theoretical estimations are inaccurate, requiring experimental performance verification. A single receiving coil and multiple transmitting coils were arranged not on a curved surface but on a plane of the chest. The performance test was conducted by applying the configuration in Fig 4.

The 3-axis milling machine was used to adjust the distance between the sending and receiving coils. The transmitting coil and the receiving coil are arranged to be parallel to each other and to move horizontally in the figure. The transmitting coil module was attached to the head of the milling machine and the receiving coil was placed on the bed, as shown in Fig 4a. The head of the milling machine was fixed, and the bed was moved 3 mm to the left and then the transmitting coils were aligned as in Fig 4c. At every operation, the voltage of the receiving coil was measured and then moved again. Fig 4d is a schematic circuit diagram for operating the transmitting and receiving coils and measuring voltages. In the actual performance measurement system, the transmitting coils were configured by connecting a single signal source to each of 7 toggle switches: the receiving coils were constructed by an LC resonance circuit. The resonant frequency is selected as 34 kHz, which allows adjustment and output of the MCU and is outside the audible frequency range [13]. The coils were manually made. Since the shapes did not conform to typical calculations of coil inductance, the capacitance causing LC resonance was found at experimentally selected frequencies. The highest resonance voltage was measured when 0.1 uF was applied.

**Fig 5:**
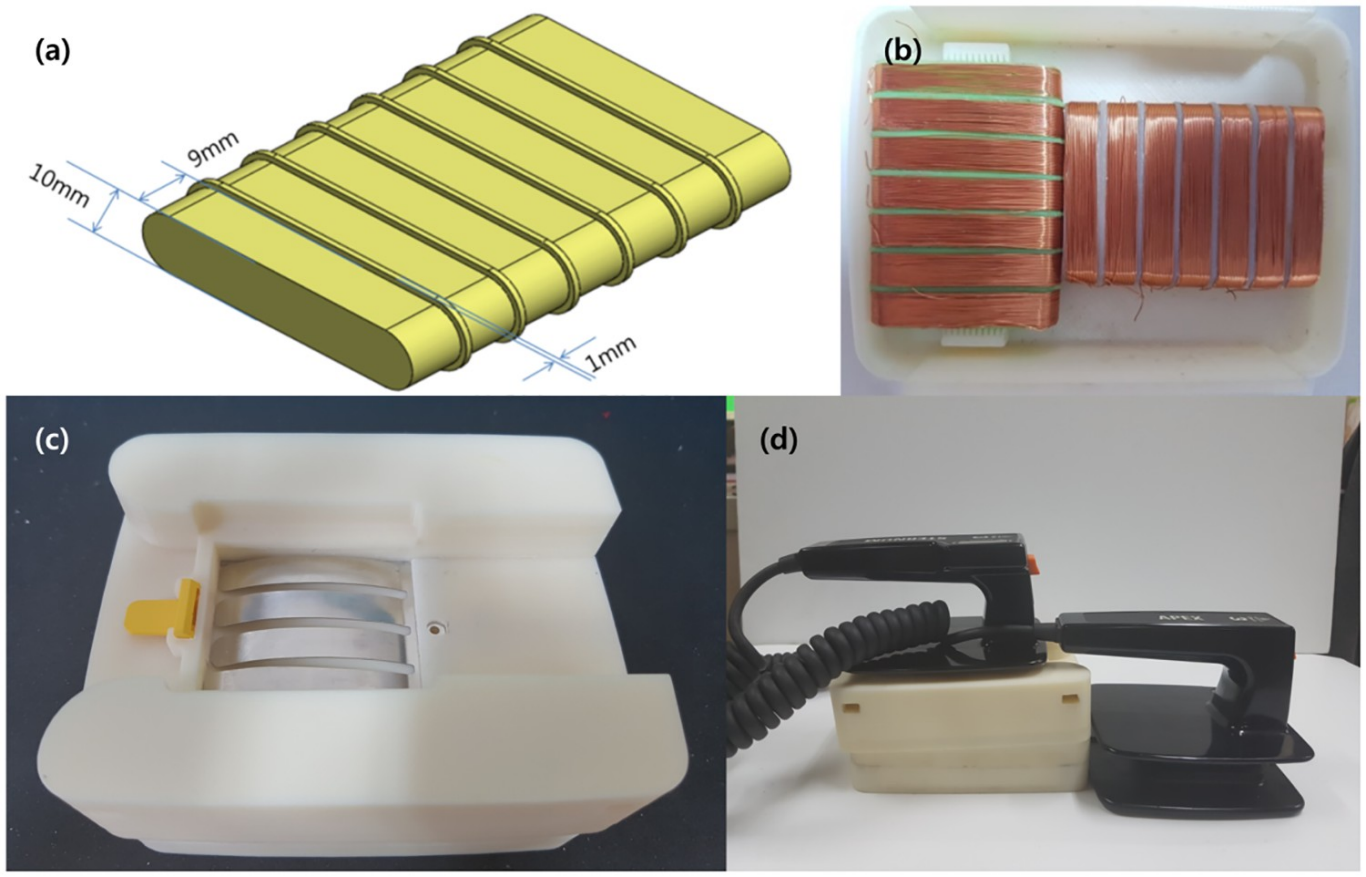
Paddle assembly for position measurement system. (a) Dimension of transmitting coil’s bobbin (b) assemble coil bobbin set into paddle box (c) assemble parts into complete part for position measurement system (d) assemble developed paddle parts substituting electrode plate of defibrillator.

The transmitting coils were arranged in the paddles and configured as shown in Fig 5. Seven transmitting coils were placed in both the transverse and longitudinal directions for detecting positions. Each coil had a diameter of 10 mm, a width of 9 mm, and 50 windings. Coil’s bobbins were modeled to set the space between coils to 1 mm and were fabricated by a 3D printer, as shown in Fig 5a. Two sets of coils were arranged in each paddle, as illustrated in Fig 5b, and were configured to detect locations both in the transverse and longitudinal directions. A mount was also modeled to connect the coils to the paddles of commercial AEDs. The mount was fabricated and assembled, as shown in Fig 5c, and was connected to paddles, as shown in Fig 5d. Detailed drawings of the paddles are included in S1 Fig.

**Fig 6:**
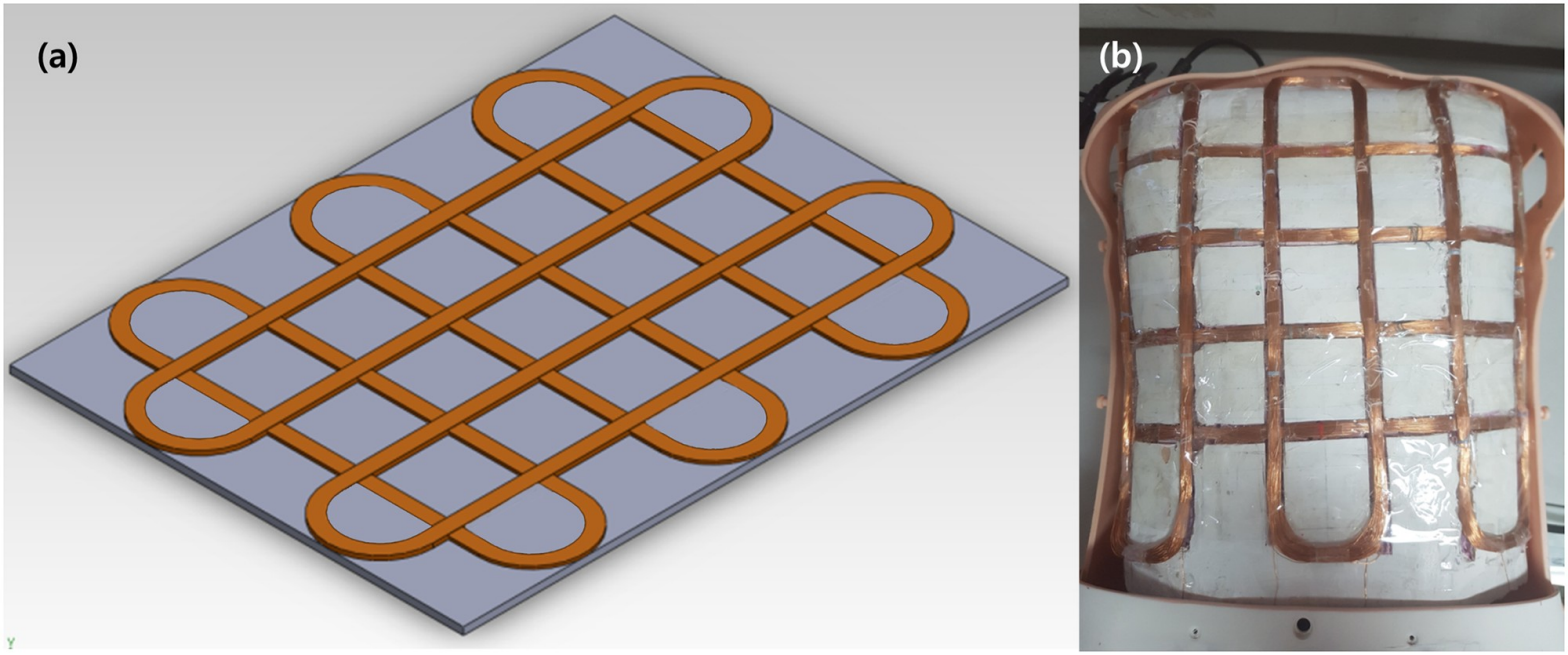
Modeling and Implemented chest manikin. (a) receiving coils array modeling using 3D CAD (b) fabricated chest manikin including receiving coils array.

The receiving coil was attached to the manikin and needed to be arranged so as to detect locations in both the transverse and longitudinal directions, like the transmitting coils. Accordingly, 3 receiving coils were placed for positioning in the transverse direction and 2 receiving coils were arranged in the longitudinal direction. To distinguish the neutral position of each receiving coil, the receiving coils were fabricated using extruded Styrofoam insulation to fit the distance between coils of both ends of a transmitting coil, as shown in Fig 6. The receiving coils of the chest mannequin were arranged and fabricated in such a way that the plane coil of Fig 6a was covered on top of the Styrofoam. Styrofoam serves to position and shapes the receiving coil along with the thoracic surface formation below the silicone skin of the chest mannequin.

**Fig 7:**
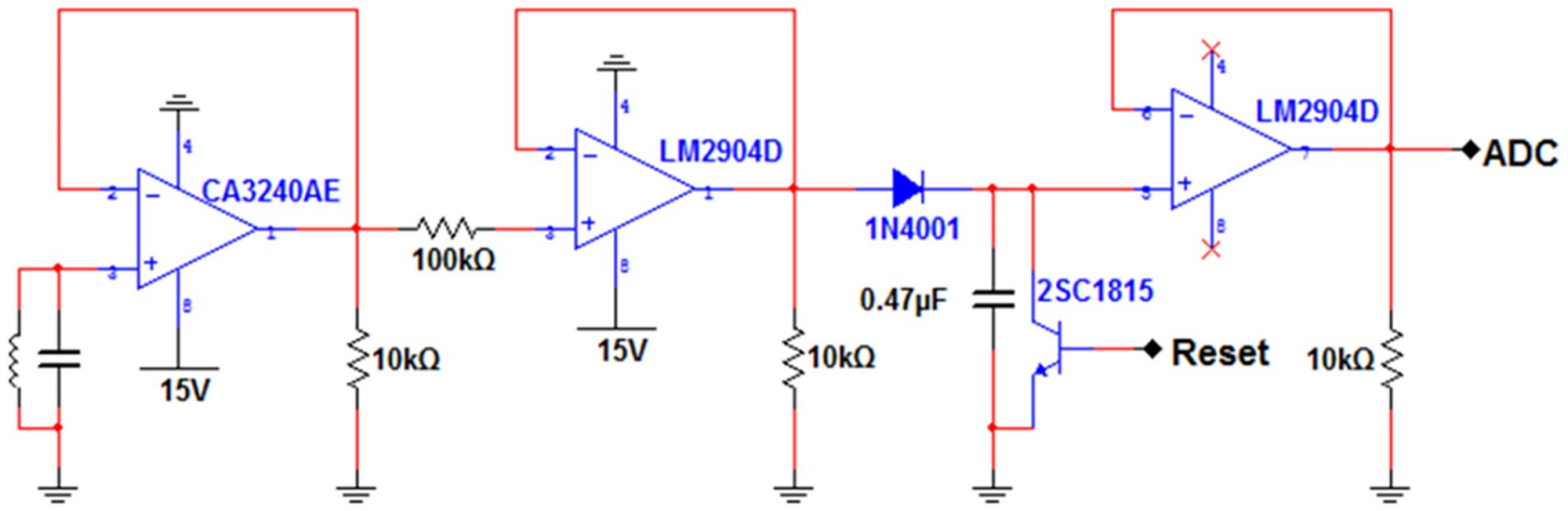
Schematic diagram of additional circuit for induced resonance. The inductor symbol in the left OPAMP part of the drawing refers to the coil placed in the chest model, and the capacitor is a capacitor whose target resonance frequency is 34 kHz. The remainder on the right is the peak hold detector schematic.

To complete a graphical user interface paddle positioning system using the above measuring system, signals had to be changed to fit the input range of the analog-to-digital converter (ADC) of MCU. Since AC signals are generated from the induced resonance coil, they change over time and have polarity. The circuit configuration in Fig 7 maximized the induced resonance of the ADC and minimized the disturbance. The operation amplifier (OPAMP) at the front end used high input impedance to configure a voltage follower circuit, thereby constructing a buffer. Then, a peak hold detector circuit was configured and only voltage peaks were detected and output by using input signals from the buffer. The output was transmitted to the ADC channel of MCU.

As shown in Fig 7, this study configured the same number (five) of the circuits as receiving coils. The circuits were connected to ADC ports of MCU. As for the coils and capacitor of the left LC-tank circuit, the optimal values of each receiving coil were experimentally determined so that induced resonances could occur at the resonance frequency of 34 kHz of the transmitting coil. The signal values thus input measured locations through the algorithm inside the MCU.

**Fig 8:**
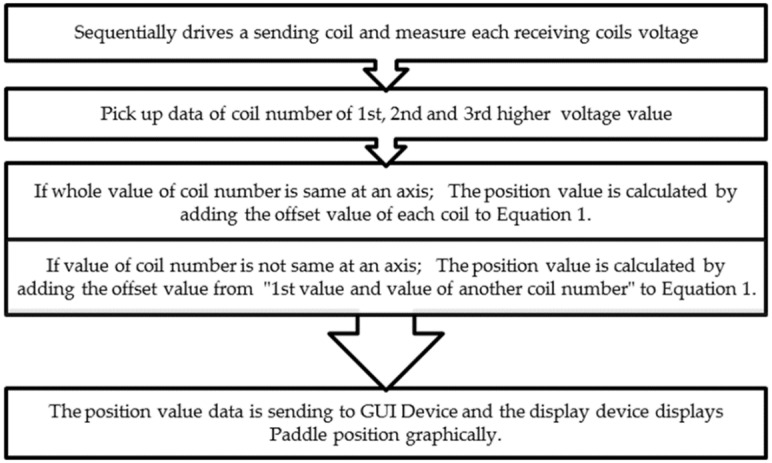
Schematic flowchart of position measuring algorithm in MCU.

Fig 8 shows the flow chart of operation. When a user presses the positioning button on the display, the request is sent to the MCU, which collects data by implementing the algorithm in Fig 8. There are two defibrillator paddles. Since the defibrillation current has polarity, paddles on the display must be distinguished. Accordingly, the positions of each paddle are measured at different times. When the positions of both paddles are completely measured, the MCU transfers the location information to the display. The received values are converted to coordinate values, and then the current paddle locations appear on the screen.

## Results

This study dealt with developing a simulator for training a defibrillation technique: the accurate positioning of defibrillator paddles. To fabricate a realistic simulator, we constructed a contactless positioning system that uses an induction coil method. Transmitting coil set consist of seven coils were arranged in both the transverse and longitudinal directions in each paddle. Three receiving coils for transverse position detection and two receiving coils for longitudinal position detection were placed on the chest manikin. Since all five receiving coils are configured to resonate at the same frequency, we have checked the performance using one receiving coil and set of seven transmitting coils.

**Fig 9:**
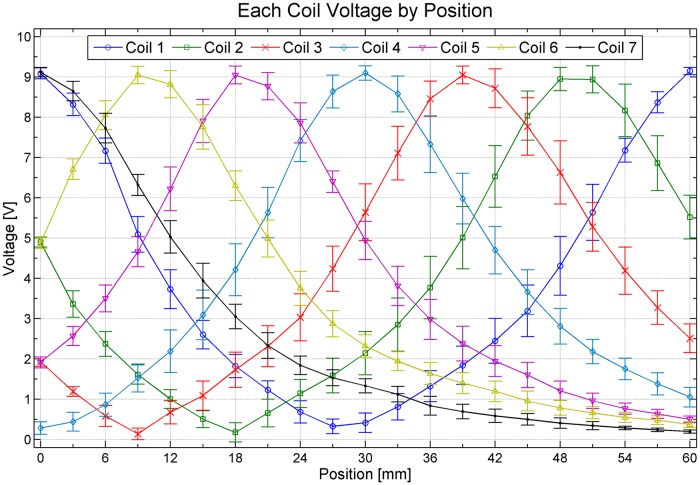
Result graph of measured voltage at Fig 7 circuit output(ADC point).

Fig 9 shows a graph of measured peak voltages. The horizontal axis indicates the locations of coils in the transverse direction and the vertical axis shows the amplitude voltages measured at each coil. The location marked 0 mm indicates that transmitting coils 1 and 7 are placed at both sides of the receiving coil (see Fig 3b). Locations mean that the bed (receiving coil) has been moved 3 mm to the left from the reference location. As the reference location implies, the highest voltage is induced by placing transmitting coils 1 and 7 over the receiving coil. When a transmitting coil becomes distant from the receiving coil, the induced voltage of the transmitting coil is smaller; the voltage of coil 4, which was farthest from both sides of the receiving coil, was the lowest.

When the receiving coil was moved in the x-direction, different coils were adjacent to the receiving coil and the corresponding voltage induced in the receiving coil changed. In addition, at 60 mm, only transmitting coil 1 was located over the receiving coil and the voltage induced by coil 1 was highest; as the distance from the receiving coil increased (that is, the coil number increased), the measurement voltage decreased.

The data in Fig 9 are voltage values. These voltages need to be transformed into locations by a formula. The physical value of the current location can be obtained using a relative voltage value of the receiving coil, which was induced by each transmitting coil based on the absolute voltages of the transmitting coils. To identify locations without detecting the phases of transmitting and receiving coils, Eq (1) was used to derive location values. For the induced voltages of each coil at one location in the measurement data table, the minimum value that left only the three highest values behind was set as a threshold; then, the measurements were subtracted from the thresholds thus determined. Next, since each coil was 10 mm from transmitting coil 1, which was on the far left, weights were applied according to coil number. The highest and second-highest voltages were compared to derive the relative location of the transmitting coil.

$$X=(7-C_{t})\times 10+(C_{t}-C_{s})\times (\frac{V_{s}}{V_{t}+V_{s}})\times 10$$ (1)

$$C_{t}=Coil\;Number\;of\;Maximum\;Voltage\;C_{s}=Coil\;Number\;of\;2nd\;Higher\;Voltage$$

$$V_{t}=Maximum\;Voltage\;Value\;V_{s}=2nd\;higher\;Voltage\;Value$$

$$※If\;C_{t}<C_{s}\;then\;C_{t}<->C_{s}\;※V_{t}=V_{s}\;then\;hihger\;Coil\;Number\;is\;C_{t}$$

**Fig 10:**
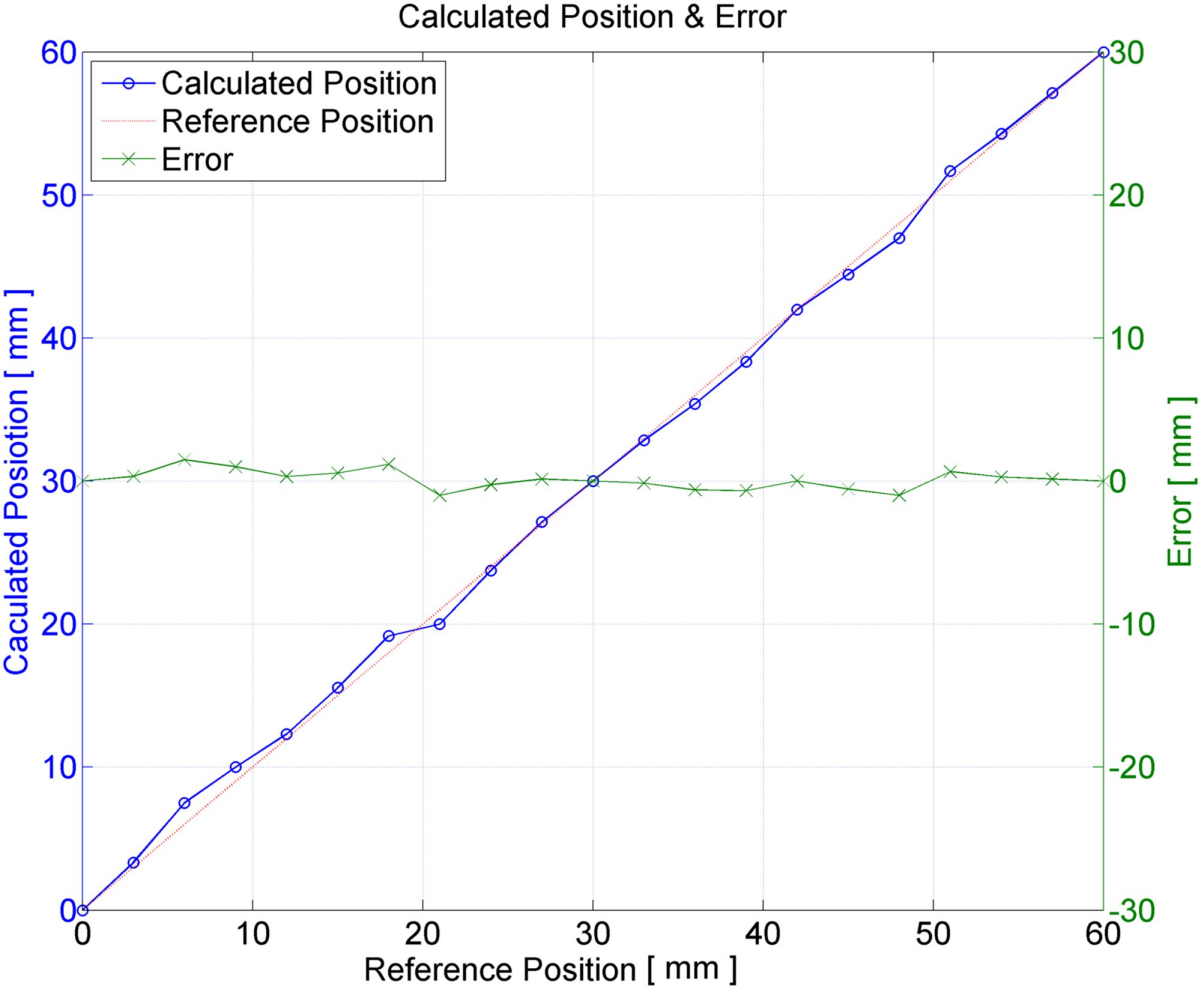
Result graph of calculated position and error. Red dotted line is ideal line and blue solid line (marked with circle) is reference versus calculated position result. And green solid line (marked with x) is position error compared with ideal position.

The derived and real locations were compared using the voltage measurement. The left side of the y-axis of the graph in Fig 10 indicates calculated locations obtained by physically moving the coils, operating the transmitting coils, and then measuring the voltages of the receiving coil and applying these measurements to an equation. The right side of the y-axis shows errors. Other than the area from the reference location (where transmitting coils 1 and 7 were placed over the receiving coil) to 10 mm, there was no significant difference from the real locations. The average error for all locations was 0.0891 mm, with a standard deviation of 0.6611 mm. The maximum error, 1.5 mm, occurred at the 6 mm point.

**Fig 11:**
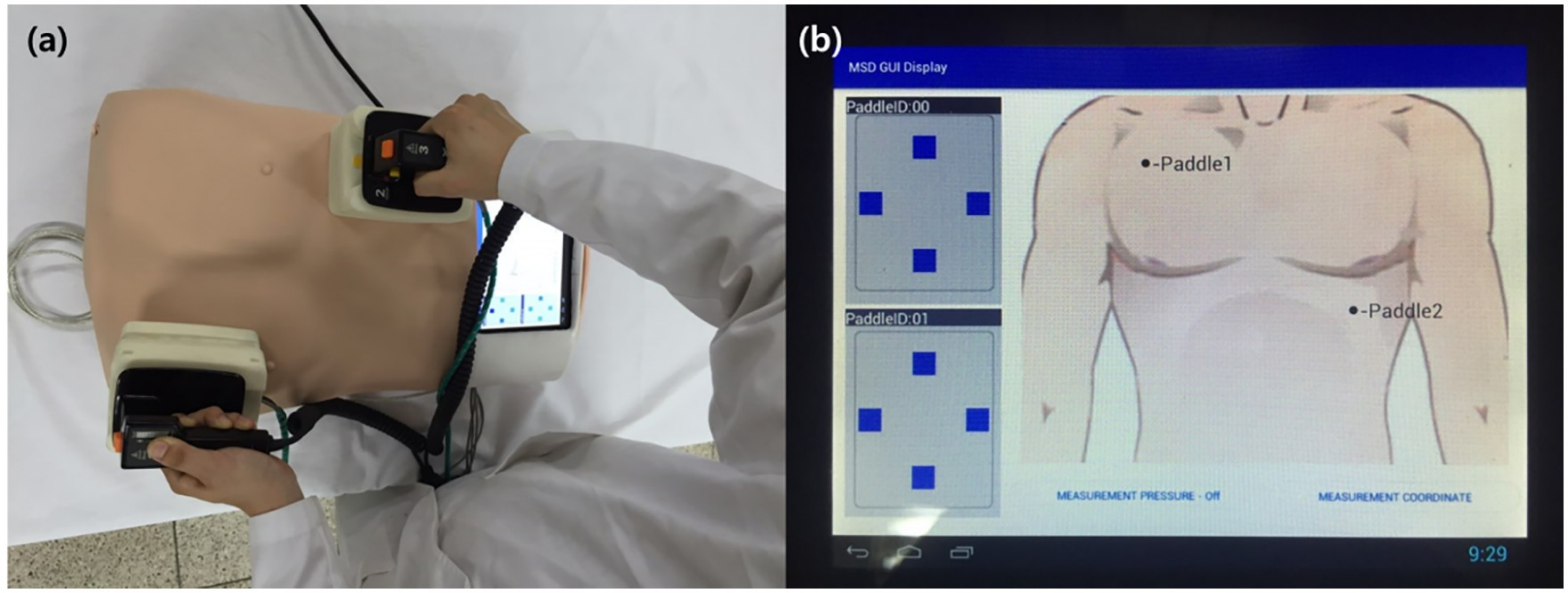
Fabricated whole defibrillation simulator with function of position measurement system. (a) demonstrate fabricated defibrillation simulator (b) display screen shows position measuring result.

Fig 11 illustrates the chest model of the complete simulator, the paddle demonstration, and the display. Instead of the electrode plates of commercial paddles, our location-measuring paddles were attached. The current locations of the paddles were measured by pressing the display positioning button. If the locations cannot be detected, an error message is displayed. The image on the left side of the display, which has not been mentioned in this study, shows another function to be added: the status of an instrument measuring the pressures of the paddles.

## Discussion

**Fig 12:**
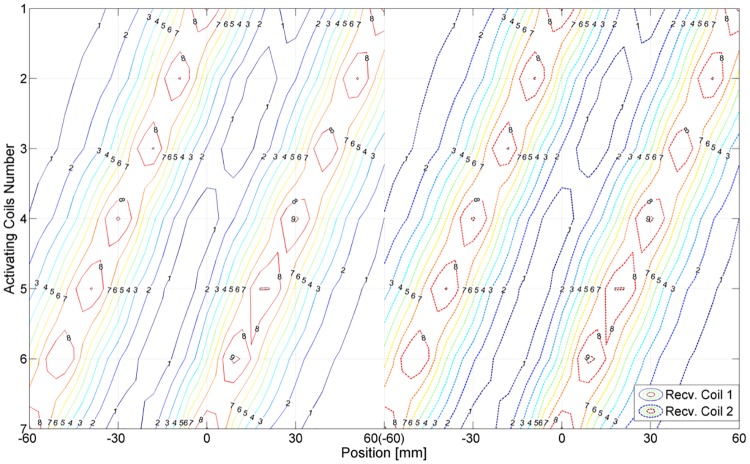
Contour graph to find errors. It is the result of combining two single coil graphs separated by the same distance as the developed system.

This study’s outcome included errors that occurred at some undetectable locations; these errors were found to mainly occur around the midpoint of the two receiving coils (where transmitting coils 1 and 7 were located over different coils). When the configured circuit, IC, MCU, and software were checked for normal operation, no problems were found. Ultimately, we found that when two coils had similar voltages, an error occurred not at the midpoint but on its periphery. From this, one assumption can be made: the receiving coil was designed to have a width that was the same as the distance between transmitting coils 1 and 7; the clearance of each receiving coil was the same as the distance from a transmitting coil. However, since the system, which had a symmetrical configuration, did not require measurement of the phase of the induced voltage, locations could not be identified. The contour graph of Fig 12 was drawn from measurements of a single coil. This graph shows the problem in the arrangement of the system with two coils. Point (a) shows the case where transmitting coils 1 and 7 are placed over different receiving coils. Points (b) and (c) indicate the case in which transmitting coil 4 was placed over each receiving coil. Point (d) indicates different receiving coils but the same location as (b). The problem occurs near point (a). Since locations are identified by top three highest values measured at the receiving coil and calculated by top two highest values, errors are expected. One alternative for solving this structural problem is adopting the structure of a Vernier caliper, which measures a length by attaching a scale to the caliper. The basic Vernier caliper divides 9 gradations of the main scale into 10 equal lengths on the caliper scale. If this structure is applied to the receiving and transmitting coils of the measuring instrument, the above problem could be solved. This issue will be dealt with in future work.

## Supporting information

S1 Fig Paddle 3D modeling drawing. (PDF)
