# Supplementary figures and images for: Development of defibrillation simulator with LC-Tank type inductance coupling position measurement system
Source: PLoS One. 2019 Mar 29;14(3):e0214576. doi: 10.1371/journal.pone.0214576 (PMC6440618; doi:10.1371/journal.pone.0214576)

# 1 Supporting Information

2 S1. Paddle 3D modeling drawing

3 Part1

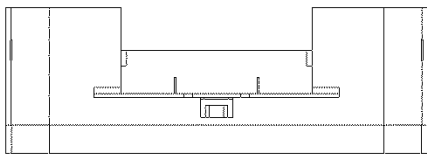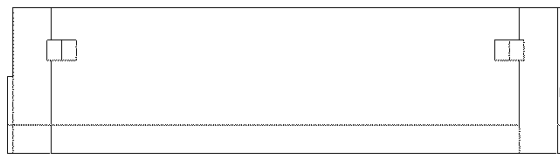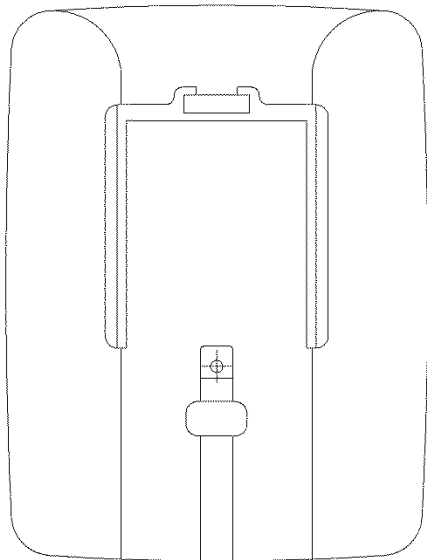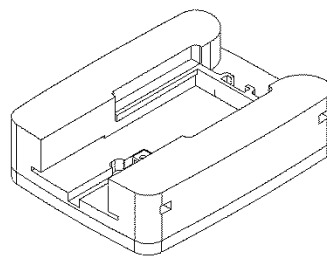

4

5

6

7 Part2

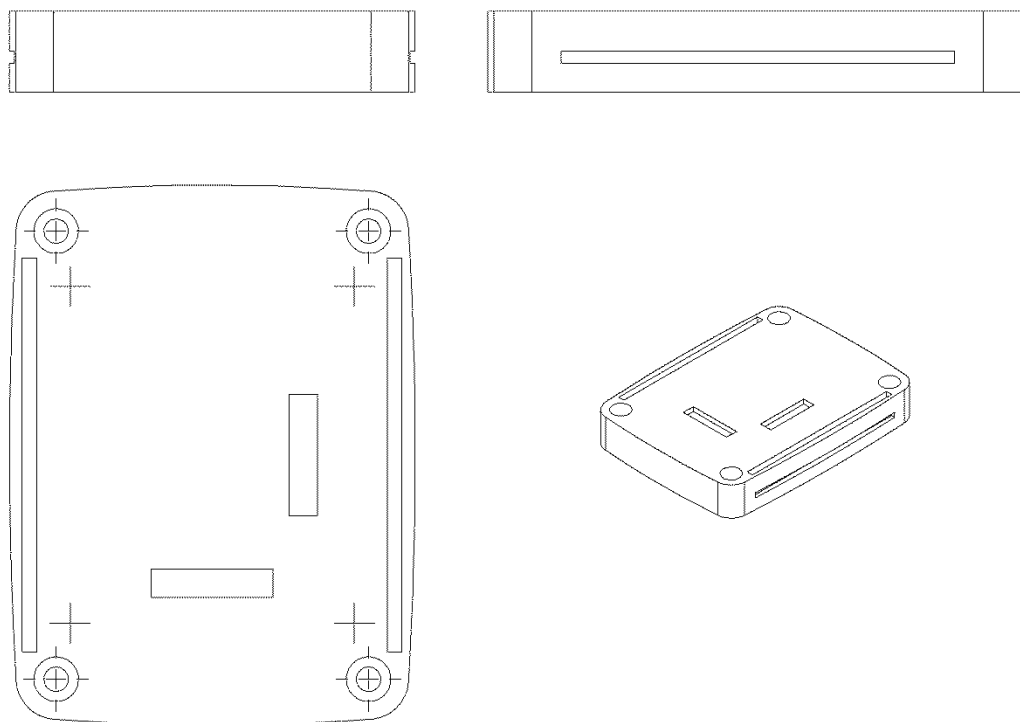

8

9

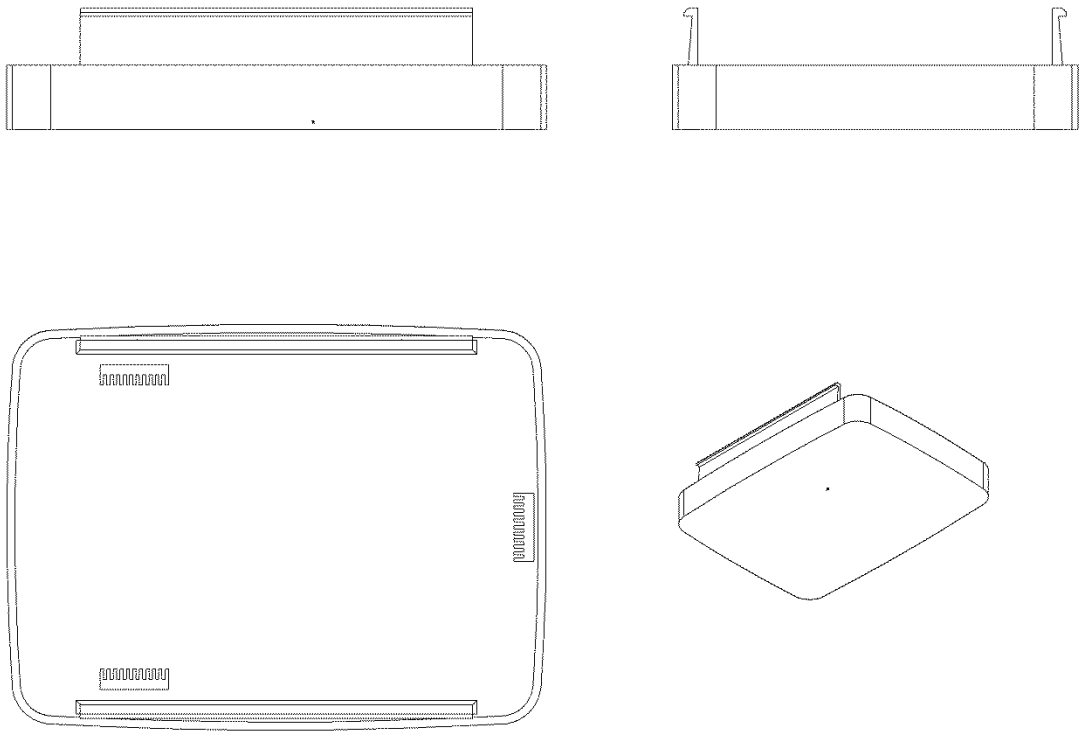

## 13 Assembly

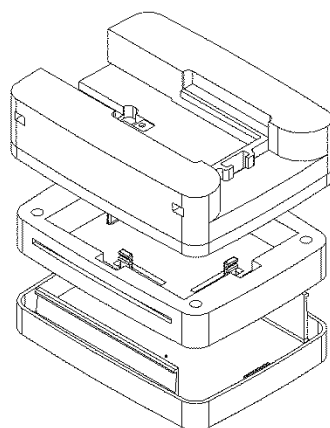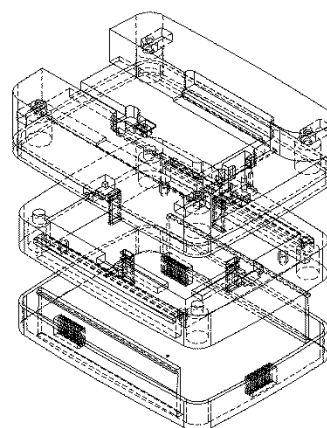

Supplement: S1 Fig — (PDF) [file pone.0214576.s001.pdf]
